# Recurrent Idiopathic Thrombocytopenic Purpura Associated With Splenosis: A Case Report

**DOI:** 10.7759/cureus.80622

**Published:** 2025-03-15

**Authors:** Jose L Mejia, Luis Mejia Sierra

**Affiliations:** 1 Surgery, WellSpan Ephrata Community Hospital, Ephrata, USA; 2 Vascular Surgery, Northwell Health, Long Island, USA

**Keywords:** bleeding, platelets, purpura, splenosis, thrombocytopenia

## Abstract

Immune thrombocytopenic purpura is a bleeding disorder in which the immune system develops antibodies against its cells, such as platelets, causing purpura and hemorrhagic episodes. The problem can be primary or idiopathic and secondary to other conditions, such as certain drugs and autoimmune conditions like lupus erythematosus. In children, spontaneous remissions are not uncommon, but they are rare in adults, in whom, after a period of medical treatment, splenectomy is necessary. Recurrence after surgery requires extensive workup, and although not quite common, remnants of the spleen or splenules should be suspected as one of the causes. The present case report relates to a recurrent disease episode several years after surgery, its management, and evolution after reoperation.

## Introduction

Immune thrombocytopenic purpura (ITP) is typically a diagnosis of exclusion. It may be idiopathic but can be triggered by various other factors, such as HIV, lymphoma, lupus, rheumatoid arthritis, *Helicobacter pylori *infection, COVID-19, pregnancy, drug use, or certain vaccines like measles, mumps, and rubella (MMR). Once a diagnosis is confirmed, a therapeutic plan must be implemented, especially for symptomatic patients. Pharmacologic treatment is the first line of approach, while splenectomy has been relegated to a secondary option [1,2].

ITP is classified as newly diagnosed if it occurs within three months after diagnosis, persistent if the symptoms last between three and 12 months, and chronic if the symptoms persist beyond 12 months. The newly diagnosed form is more common in children, with an incidence of 1.9-6.4 per 100,000 per year, without sex discrimination. Chronic ITP is more prevalent in adults, with an incidence of 3.3 and a female predilection two to three times higher than males [1].

Bleeding is the primary symptom but is rare unless the platelet count falls below 30,000/MCL. These patients typically present with cutaneous and mucosal bleeding, while more severe complications such as hemarthrosis, hematuria, gastrointestinal bleeding, and intracranial bleeding usually occur when the count drops below 10,000/MCL. Additionally, fatigue is a common symptom in adults (58%) and children (22%). Although uncommon, thrombosis can be present due to the release of procoagulant microparticles by activated platelets [2].

## Case presentation

**Figure 1:**
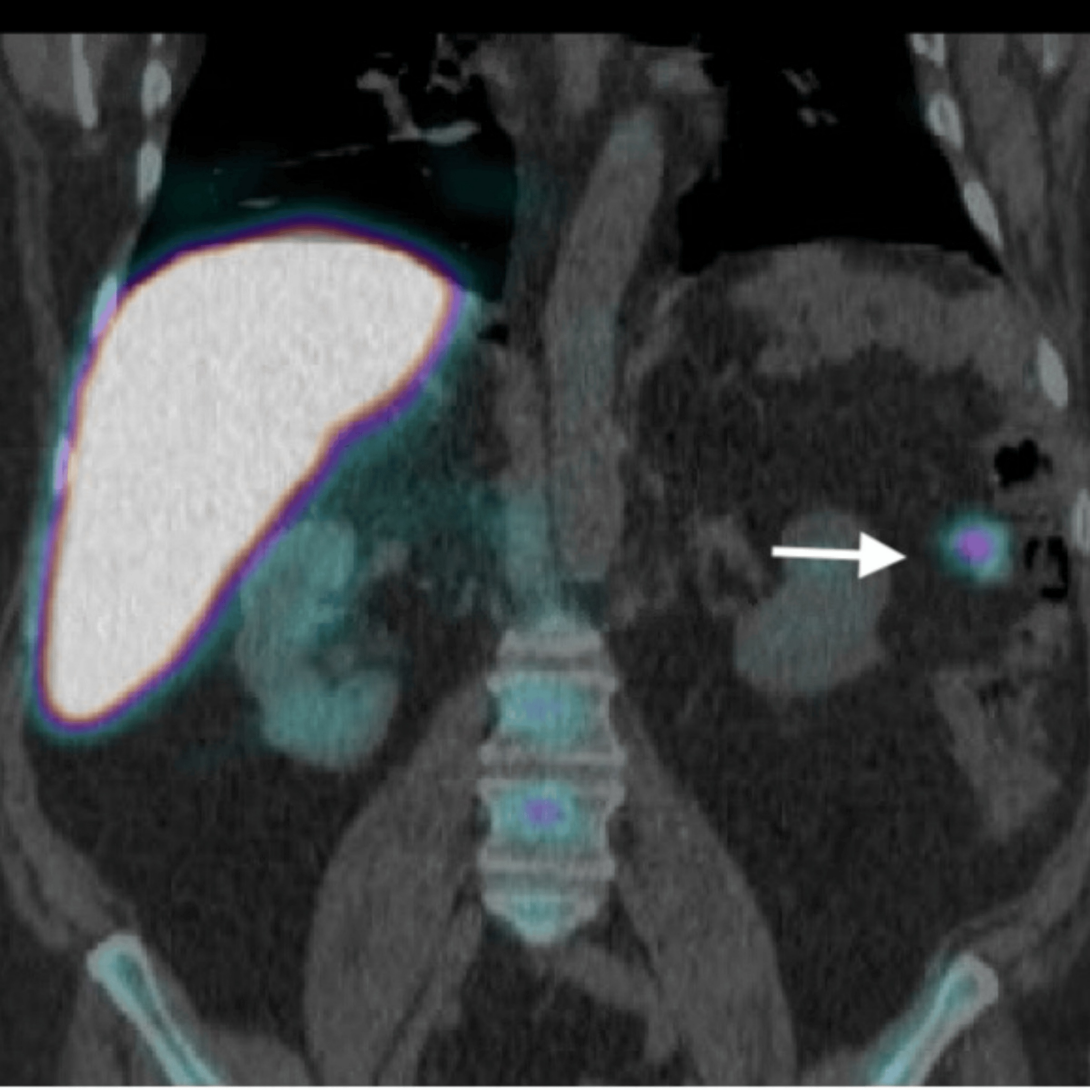
Splenosis (arrow: splenic tissue) The positron emission tomography (PET) scan image shows remnant splenic tissue located in the splenic bed.

**Figure 2:**
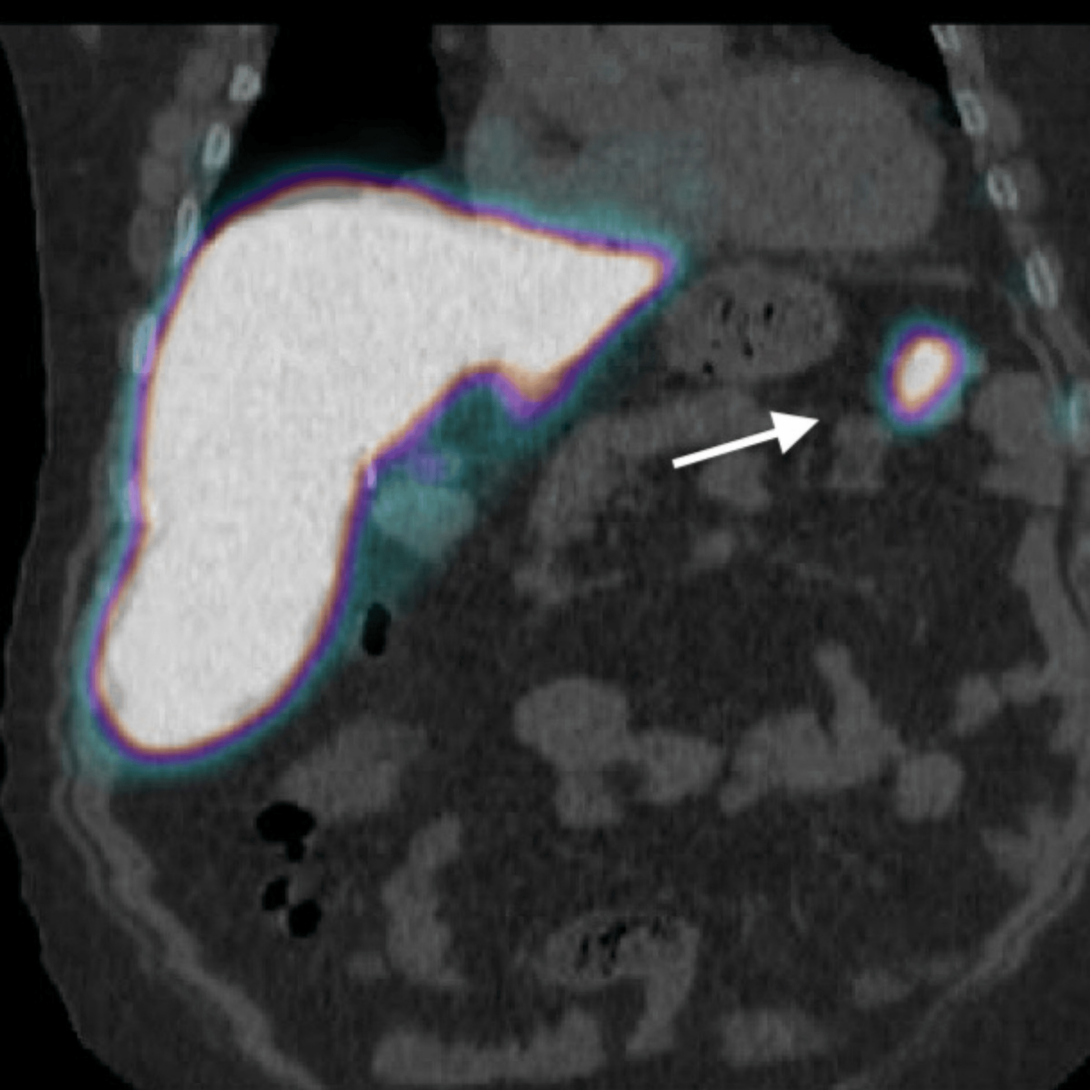
Splenosis (arrow: splenic tissue) The positron emission tomography (PET) scan image shows remnant splenic tissue near the tail of the pancreas.

We present the case of a 47-year-old male patient diagnosed with ITP in 2011. His comorbidities are autism, diabetes mellitus type II, and obstructive sleep apnea. Clinically, he presented for evaluation of mucosal bleeding from the mouth, epistaxis, generalized ecchymosis, and asthenia. Initially, the patient received treatment with steroids and intravenous immunoglobulin (IVIg), followed by rituximab. Unfortunately, the patient did not respond as expected and underwent an open splenectomy four months later. Although an initial positive response was observed, he experienced multiple recurrences. In 2015, eltrombopag was used, leading to favorable results for a short period. However, a relapse occurred later, requiring subsequent hospital admissions for medical management. The patient's platelet count dropped to as low as 1,000/MCL. In 2023, the patient underwent re-evaluation with imaging, including a CT scan of the abdomen, which revealed postoperative changes but no apparent signs of splenosis. Subsequent positron emission tomography (PET)-CT imaging demonstrated at least four hyper-enhancing areas in the left upper quadrant (Figures 1, 2). Based on these findings, he was referred for possible re-exploration.

A few days before the procedure, the patient received an IVIg infusion, which successfully corrected the platelet count to normal levels. Subsequently, an exploratory laparotomy was performed, revealing a 5-millimeter lesion at the previous abdominal wall incision site, as well as two lesions measuring approximately 3-5 cm at the tail of the pancreas. Another 2-cm elongated lesion was found in the splenic fossa, and a well-encapsulated 4-cm mass was discovered in the perinephric fat. The latter lesion was believed to be an accessory spleen. Intraoperative pathology analysis was positive for splenic tissue. The procedure was completed without complications, and the patient remained hospitalized for five days. The platelet counts normalized, and the patient's counts remained stable at a two-week follow-up.

## Discussion

Multiple mechanisms contribute to the development of this disease. Patients with ITP produce antiplatelet IgG antibodies, rarely IgM or IgA, which bind to platelet glycoproteins, marking them for phagocytic breakdown in the spleen and liver. However, up to 40% of patients do not have detectable antibodies, suggesting other mechanisms such as antigen cross-reactivity (mimicry), somatic mutations, or problems with eliminating auto-reactive B-cell clones. Plasma cells, which are known to be increased in this pathology, secrete autoreactive antibodies. Still, as mentioned earlier, many patients do not have detectable plasma and/or platelet-bound antibodies, indicating the involvement of other causes such as T-cell imbalance. Higher reactivity of T helper cells against platelets has been observed, and CD8 T cells can directly lyse platelets. Furthermore, CD8 T cells can accumulate in the bone marrow, inhibiting thrombopoiesis [1,2].

Antigen-presenting cells, primarily dendritic cells, are also impaired in ITP, leading to abnormal self-antigen presentation and the production of pathogenic antibodies. Research has revealed that anti-platelet autoantibodies and T cells target megakaryocytes in the bone marrow, resulting in impaired maturation and reduced platelet production in ITP. Toll-like receptor-mediated recognition of infection agents has also been associated with ITP. Approximately two-thirds of patients have documented autoantibodies that inhibit megakaryocyte maturation and induce apoptosis [1,2].

Diagnosis of ITP is primarily based on exclusion and relies on a thorough medical history and physical examination. It is crucial to rule out secondary causes, and a platelet count below 100 × 10^9/L indicates this condition. In addition to a complete blood count (CBC), a peripheral blood smear, antiplatelet antibody testing (with a sensitivity of 53%), and a bone marrow biopsy are essential components of the diagnostic workup [2].

The initial ITP treatment involves corticosteroids, IVIg, and anti-RhD immunoglobulin. However, data suggests that only 10-30% of patients achieve sustained remission with steroid therapy. Second-line agents with robust evidence include rituximab, which inhibits B cells from producing antibodies, and thrombopoietin receptor agonists such as eltrombopag or romiplostim, which act on the megakaryocyte receptor. The use of immunosuppressants like azathioprine, cyclosporin A, or cyclophosphamide has less robust evidence supporting their efficacy [1,2].

Fostamatinib is a spleen tyrosine kinase inhibitor recently approved for treating chronic ITP in patients who have not adequately responded to at least one prior line of therapy. Splenectomy, once a primary treatment option, has been less frequently employed in recent years due to advancements in medical therapy. However, in cases of treatment failure, it is essential to consider surgical intervention [1,2].

Splenosis is a benign condition characterized by the heterotopic viable auto-transplantation of splenic tissue to various body areas. It can occur after trauma or splenectomy and may be observed in the setting of recurrent ITP. Abdominal splenosis is often an incidental finding, and CT and magnetic resonance usually allow for a confident diagnosis. The typical enhancement that parallels the spleen is a valuable hallmark of splenosis. Splenic implants lack contrast uptake in the hepatobiliary phase and show high signals at high b-values on diffusion-weighted images [3,4]. Additional modalities, such as PET-CT, can assist in identifying suspicious areas in the appropriate clinical setting [5,6].

## Conclusions

ITP is a rare autoimmune disorder characterized by a low platelet count, which can lead to increased bruising, bleeding, and other associated complications. The exact cause of ITP remains unclear, with multiple potential etiologies contributing to its onset. However, even in the case of recurrence, it is a condition that primarily requires pharmacologic treatment to manage platelet counts and prevent bleeding episodes. However, when medical treatments fail or the patient experiences chronic or severe forms of the disease, surgery may become a necessary consideration. Surgical interventions, such as splenectomy, are typically reserved for cases that do not respond to pharmacologic therapy.

Splenectomy and re-exploration can be performed through various approaches, including open, laparoscopic, and robotic techniques, each with its advantages and limitations. The choice of surgical method often depends on the patient's condition, the surgeon’s expertise, and available resources. Recently, there has been interest in incorporating radio-guided technology to assist in precisely identifying and removing the remnant of splenic tissue, which could represent a promising advancement in surgical treatment. This new tool may enhance the procedure's effectiveness, particularly in complex cases where conventional methods might pose challenges.
